
JOURNAL INFORMATION
==============================
NLM Title Abbreviation: Tex Med J (Austin)
NLM Journal ID: 21130590R

Texas Medical Journal

ISSN: 0892-8495
Publisher: Biomedical Journal Digitization Project

ARTICLE INFORMATION
==============================
PMCID: PMC9642149
Article version: 1
Subjects: Books and Magazines

Herself

Talks with Women Concerning Themselves

Print publication date: 1911 Nov
Volume: 27
Issue: 5
First page: 193
Last page: 193
License: This work is free of known copyright restrictions. For more information, please see PMC Back Issue Digitization.
License URL: https://creativecommons.org/publicdomain/mark/1.0/

==============================

---

### Books and Magazines.

HERSELF.—Talks with Women Concerning Themselves. By Dr. E. B. Lowry, author of "Confidences," "Truths," etc. Forbes & Company, Chicago. Price $1. 1911.

This book contains truths vitally important to every woman. The health and happiness of mothers and their children depend upon their knowledge of the facts here given with great clearness and conciseness by a physician of national reputation in the scientific care of women.

---

### Publisher's Department.

IN NERVOUS EXCITEMENT.—The principal indications for Peacock's Bromides are, of course, Epilepsy, Uterine Congestion, Headache and all Neuroses. Being a safe and certain nerve sedative, it will be found a most valuable aid whenever the mental functions are overtaxed, producing insomnia. Peacock's Bromides does not compel sleep, like hypnotics, but by allaying the existing nervous excitement, whether due to mental strain, worry or anxiety, it promotes sleep in a normal manner. Unlike the effects from hypnotics, the patient awakens refreshed, with a clear head, and does not suffer from unpleasant sequelae the following day. The overstimulation of the cerebral functions from alcohol yields promptly to the soothing action of this preparation.

---

AN IDEAL PURGATIVE MINUS CATHARTIC INIQUITIES.—This elegant product—Prunoids—represents a real advance in the therapy
